# AnglerFish: a webserver for defining the geometry of α-helices in membrane proteins

**DOI:** 10.1093/bioinformatics/btw781

**Published:** 2017-01-12

**Authors:** Matthew Colledge, B A Wallace

**Affiliations:** Institute of Structural and Molecular Biology, Birkbeck College, University of London, London, UK

## Abstract

**Summary:**

Integral membrane proteins that form helical pores and bundles constitute major drug targets, and many of their structures have been defined by crystallography and cryo-electron microscopy. The gating of channels and ligand binding of transporters generally involve changes in orientation of one or more the constituent helices in the structures. At present there is no standard easily accessible means for defining the orientation of a helix in a membrane protein structure. AnglerFish is a web-based tool for parameterising the angles of transmembrane helices based on PDB coordinates, with the helical orientations defined by the angles ‘tilt’ and ‘swing’. AnglerFish is particularly useful for defining changes in structure between different states, including both symmetric and asymmetric transitions, and can be used to quantitate differences between related structures or different subunits within the same structure.

**Availability and Implementation:**

AnglerFish is freely available at http://anglerfish.cryst.bbk.ac.uk. The website is implemented in Perl-cgi and Apache and operation in all major browsers is supported. The source code is available at GitHub.

**Supplementary information:**

Supplementary data are available at *Bioinformatics* online.

## 1 Introduction

Integral membrane proteins make up ∼30% of the human genome (Arinaminpathy *et al.*, 2009); many of these are comprised of transmembrane helical segments whose alignment with respect to each other control the translocation of ions or molecules across the membrane.

Despite the functional importance of transitions between open and closed states of channels, or outward and inward facing states of transporters, there is no universal system or tool for defining the angles associated with helix movement underpinning membrane protein conformational changes. Such changes tend to be quantified indirectly by differences in pore diameter (Smart *et al*., 1996) or as changes in ϕ and ψ angles at individual residues (McCusker *et al.*, 2012). While those approaches can inform on some features associated with gating by comparisons of ‘open’ and ‘closed’ structures, AnglerFish can be used to provide alternative quantitative and visual information about changes associated with the helical geometry. AnglerFish provides an easy-to-use webserver for defining the angles of transmembrane helices within either a symmetric or asymmetric structure, and can be used to quantify differences for related proteins in different functional states. The advantages of this approach are illustrated in the examples included in the Supplementary Information (Supplementary Figs S1–S3 and Supplementary Tables S1–S3).

## 2 Definitions of parameters

AnglerFish models a helical bundle as a rotationally symmetric arrangement of helices around a central pore of any size. The orientations of the helices are defined by two angles, tilt and swing, which describe the helical axis relative to the pore axis (the axis of rotational symmetry). The pore axis is defined as the normal to a plane containing the same residue number from each helix.

**Fig. 1.**
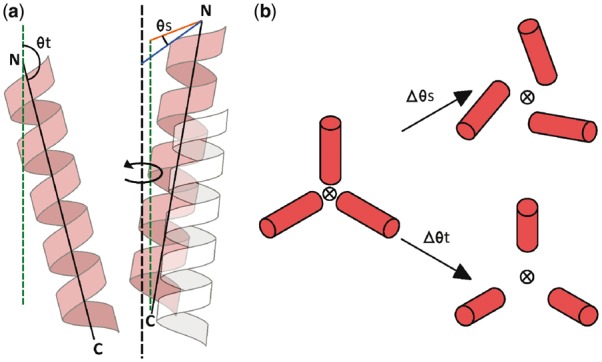
(**a**) Definitions of tilt (θt) and swing (θs): The axis of rotational symmetry (pore axis) shown as a black dotted line. N and C indicate the N-terminal and C-terminal ends of the helices, respectively. The green dashed lines are parallel to the pore axis to indicate the geometry more clearly, and the blue and orange lines are perpendicular to the pore axis. (**b**) The effects of changes in tilt and swing, as shown from above. The pore axis is indicated by the circled cross and the helices are depicted as cylinders

Tilt is defined as the angle between the pore axis and the helix axis (θt, Fig. 1a). Changes in θt can dilate a pore in the manner of limbs of an umbrella as it opens (Fig. 1b). Swing (θs, Fig. 1a) is the angle perpendicular to the pore axis with its vertex at the N-terminal end of the helix axis, with one side (blue line) defined by the line intersecting the pore axis and the other side (orange line) connecting the component of the helix axis perpendicular to the pore axis. Changes in θs can dilate a pore in a similar fashion to double doors opening in opposite directions (Fig. 1b). As the pore axis is only dependent upon the protein coordinates, θt and θs are independent of the orientation of the protein within the membrane. (i.e. the planes representing the lipid bilayer are not taken into account for the calculation of θs and θt).

## 3 Usage

AnglerFish is a web-based tool where users upload the file of interest (in protein data bank (PDB) format (Berman *et al.*, 2000)) and enter the residue numbers of the ends of the transmembrane helix of interest. In order to maintain the symmetry required for axis definition, the PDB file must be of a homo-multimer with each of the different chains containing corresponding residues numbered in the same way.

The residue number used to define the pore axis is determined by the programme: it considers each residue number along the helix and selects the one such that residues of that number in different chains lie closest to a flat plane. The ends of the alpha helix are defined as averages of the Cα atom coordinates of the first and last 4 residues within the helix, and the helix axis is defined as the line that connects the two ends.

The user is provided with the tilt and swing angles for the defined helix in each of the polypeptide chains in the file and an indication of which residues were used to define the pore axis. There is also an option for the user to manually select the residue to use for pore axis definition, if they prefer. If the calculation fails, a warning page is displayed detailing any co-ordinates specified from the input that could not be found within the file. Under the ‘advanced options’ tab, there are extra fields for ‘model number’ to use when the coordinates file contains multiple structures. The optional ‘chains to use’ field can be used to specify which of the chains to include in the calculation, in case the file contains chains that do not comprise the pore.

The Supplementary Information shows examples of uses of AnglerFish on several published structures to probe different states or asymmetries within channel structures: Example 1 shows the gating of the NaK channel, which involves the induction of a kink (Shi *et al.*, 2006; Alam and Jiang, 2009). The angles between the open and closed structures differ little for the pre-kink helix but substantially more for the helix following the kink. In addition, this example shows the complementarity of AnglerFish with a method that defines the geometry of the central pore (Smart *et al*., 1996). Example 2 compares the angles in the transmembrane helices of the open and closed structures of the GLIC receptor (Sauguet *et al.*, 2016). AnglerFish shows that the structural rearrangement in gating is dominated by helix 2 changing swing by 85.6°. A change in tilt also occurs but the difference is minimal. This illustrates the importance of calculating both the swing and tilt parameters. Example 3 illustrates the different tilt and swing angles for the gating helix of the voltage-gated sodium channel pore NavMs in the asymmetric partially open (McCusker *et al.*, 2012) and the symmetric fully open pore (Bagneris *et al.*, 2013). This shows that the S6 helix of chain A of the partially open pore differs from the S6 helices in the other chains by being more tilted; comparisons with the fully open symmetric pore, show that chain A is the monomer that is the most similar to all the chains in the fully open pore.

## 4 Conclusions

AnglerFish provides a useful tool for parameterising helical geometry in membrane proteins, especially for defining structural differences associated with conformational changes. It can be used to define the orientation of a helix about a defined axis in the protein, and can be used on multimeric proteins to probe both symmetric and asymmetric changes that occur in different subunits. It therefore provides a novel quantitative means of defining parameters associated with predominantly helical membrane proteins, such as channels and transporters. AnglerFish is available at http://anglerfish.cryst.bbk.ac.uk, and the source code is available in GitHub (ID number: 23099800).

## Supplementary Material

Supplementary Data
