# Supplementary material for: AnglerFish: a webserver for defining the geometry of α-helices in membrane proteins
Source: Bioinformatics. 2017 Jan 12;33(8):1233–4. doi: 10.1093/bioinformatics/btw781 (PMC5860525; doi:10.1093/bioinformatics/btw781)
Supplement: Supplementary Data [file btw781_supp.pdf]

Supplementary information

AnglerFish: A Website for Defining the Geometry of α-Helices in Membrane Proteins

Matthew Colledge and B.A. Wallace

Birkbeck College, University of London

Example 1: Identifying the kinked helix in the NaK channel

The sodium and potassium conducting (NaK) channel is a homotetramer (Shi et al, 2006) for which open and closed structures available (Alam and Jiang, 2009). It is gated by changes around a hinge residue, glycine 87, in the pore-lining helix, which induce a kink, leading to opening of the pore. To parameterise (Table S1) and visualise (Figure S1a) the kink in the NaK channel, tilt and swing values were calculated for the helical segments before and after the kink in the open and closed structure (PDBIDs 3e83 and 2ahy, respectively). The largest differences of tilt (22.6° and 60.3° [green overlay], respectively, in the two structures) are before and after the separate helices are placed between residues 87 and 88, showing this is the position of the kink. It should be noted that when comparing helical segments before and after a kink, only the tilt should be considered as the swing is affected by the proximity of the helix to the centre of the pore, so will be different even for a completely straight helix. Comparison of the section of the helix after the kink with the corresponding section in the closed structure shows the opening is associated with a change in tilt of 33.6° and a change in swing of 31.6° [red vs. green overlays]. In comparison, analysing the internal surface (Figure S1b) using HOLE (Smart et al, 1996) indicates the dilation of the internal pore, but as the kink occurs at a different location to the constriction, the two methods are highly complementary.

Supplementary Table 1: Tilt and swing values for the NaK channel calculated using residue 45 to define the axis.

Structure	Helix Residues	Chain								Average	
		A		B		C		D			
		Θt	θs	θt	Θs	θt	θs	θt	θs	θt	θs
2ahy closed	74-102	25.9	22.2	26.2	21.2	25.9	22.2	26.2	21.2	26.1±0.2	21.7±0.6
	74-87	24.9	18.9	24.0	17.9	24.9	18.9	24.0	17.9	24.9±0.5	18.4±0.6
	88-102	26.4	42.3	26.9	40.7	26.4	42.3	26.9	40.7	**26.7±0.3**	**41.5±0.9**
3e83 open	74-102	44.1	37.2	44.1	37.2	44.1	37.2	44.1	37.2	44.1±0	37.2±0
	74-87	22.6	24.3	22.6	24.3	22.6	24.3	22.6	24.3	**22.6±0**	24.3±0
	88-102	60.3	73.8	60.3	73.8	60.3	73.8	60.3	73.8	**60.3±0**	**73.8±0**

Supplementary Figure S1a: Pre- and post- kink helix axes are plotted on an overlay of the 2ahy structure (one chain is depicted in red with circular capped axes) and 3e83 (one chain is depicted in green with diamond capped axes). The superposition was performed using the PyMol "super" command. The pore axis is shown as a dotted line in the left panel and a crossed circle in the right panel (90 degree view).

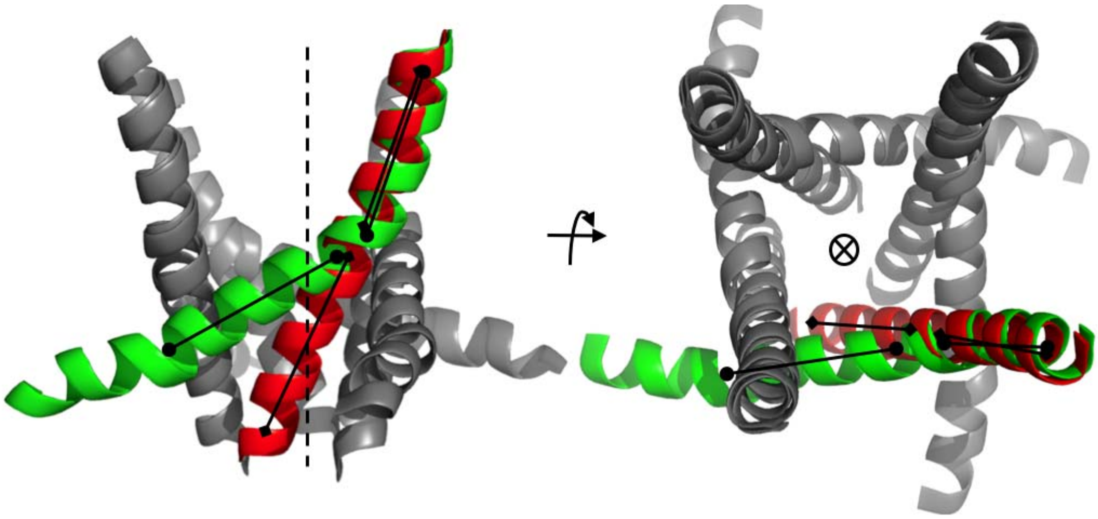

Supplementary Figure S1b: The internal surface dimensions of the NaK channel pore as calculated using HOLE (Smart et al, 1996), and depicted as a wire mesh inside the closed (PDBID 2ahy, left) and open (PDB: 3e83, right) structures, with the chains coloured as in Figure S1a.

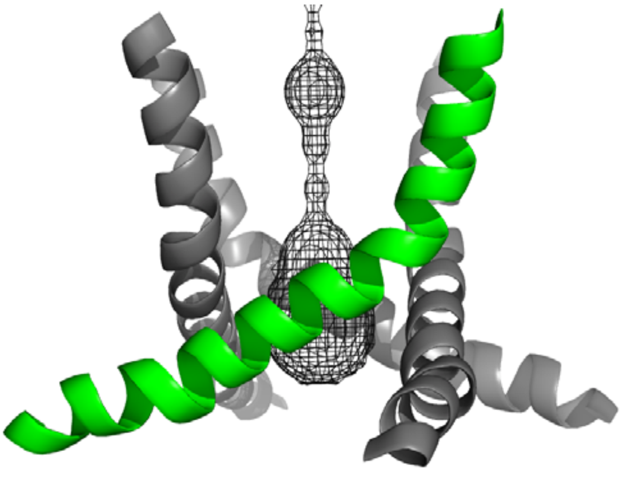

Example 2: Comparison of open and closed channel structures

The prokaryotic ligand gated ion channel (GLIC) is a homopentamer which forms a proton-gated cation channel. GLIC is of particular interest because of its homology to eukaryotic acetylcholine receptors; like acetylcholine receptors GLIC is a target of general anesthetics. Co-crystallization of GLIC with a general anesthetic produced a closed form of the channel (PDBID 4zzb) which can be used for direct comparison with the open channel (PDBID 4zzc) to investigate the structural changes involved in gating (Sauguet et al, 2016). AnglerFish was used to calculate (Table S2) and visualise (Figure S2) tilt and swing values for all four helices of the GLIC channel in the closed and open conformations. Comparisons show that the second helix (residues 221-241) in all monomers undergoes the greatest change between the open and closed structures. The difference is primarily a change in swing of the helix of 86.5°.

Supplementary Table S2: Tilt and swing values for the GLIC channel helices using residue 200 to define the axis. The average twist and swing values for the changes in helix 2 of the two structures highlighted in red and green, respectively

Structure	Helix Residues	Chain										Average	
		A		B		C		D		E			
		θt	θs	θt	θs	θt	θs	θt	θs	θt	Θs	θt	θs
4zzb	202-217	14.0	-66.0	14.9	-66.6	14.9	-66.0	15.0	-68.3	15.2	-65.0	14.0±0.5	-66.0±1.2
	221-241	170.6	73.4	170.7	72.0	170.8	73.8	170.5	71.8	170.7	74.3	**170.7±0.1**	**73.3±1.1**
	254-281	16.0	-53.0	16.8	-53.2	16.7	-52.6	16.9	-53.7	17.1	-52.5	16.9±0.4	-53.0±0.5
	285-314	161.5	135.7	161.6	136.7	161.7	137.1	161.6	135.3	161.1	135.7	161.5±0.2	136.1±0.8
4zzc	202-217	12.5	-67.8	12.4	-67.4	12.5	-67.5	12.2	-67.8	12.4	-68.9	12.4±0.1	-67.9±0.6
	221-241	174.7	157.0	174.5	157.4	174.5	158.6	174.7	161.2	174.8	159.3	**174.6±0.1**	**158.9±1.6**
	254-281	17.1	-45.1	16.0	-45.1	17.1	-44.5	16.0	-44.5	16.8	-45.3	16.0±0.6	-44.9±0.4
	285-314	160.8	146.3	160.8	146.4	160.6	146.8	160.6	147.4	160.9	146.5	160.8±0.1	146.7±0.4

Supplementary Figure S2: Helical axes plotted on the overlaid 4zzb (in red with diamond capped axes) and 4zzc (in green with circle capped axes) structures; the helices are numbered as in Table S2. Superposition was performed using the Pymol "super" command. The pore axis is shown as a dotted line and circled cross. The numbers correspond to the order of the helices from N- to C- termini.

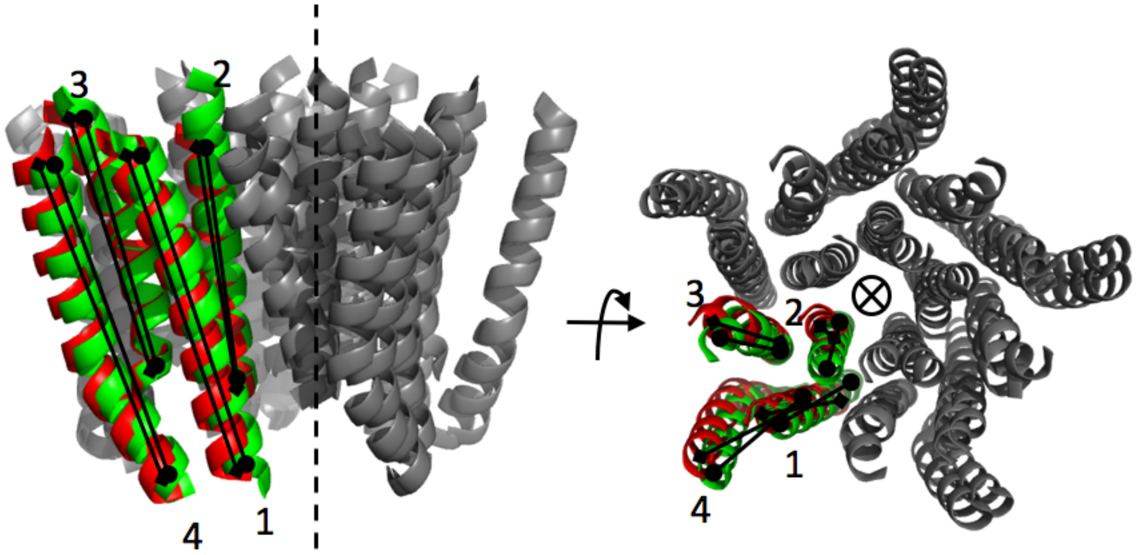

Example 3: Analysis of the asymmetric opening of the NavMs pore:

The voltage-gated sodium channel from *Magnetococcus Marinus* (NavMs) is a homotetramer and is used as a structural model for eukaryotic Navs. The first open structure sodium channel structure (McCusker et al, 2012) solved that was of the NavMs pore. This structure showed the pore to be asymmetric, with the gate partially open, as a consequence of a different psi angle in one (chain A) of the S6 helices monomers. A later structure of the NavMs pore (Bagneris et al, 2013) depicted the symmetric conformation of a fully open pore.

In order to demonstrate the asymmetry of the partially-open pore (PDBID 4f4l), tilt and swing values were calculated for each chain. Because chain A corresponded to the altered conformation, chains B, C and D were used to define the pore axis; had all chains been used, they would have skewed the orientation of the axis. Tilt and swing values for chain A were 141.0° and 27.2°, respectively, which fall outside the s.d. of the mean for chains B, C and D (142 ± 0.9° and 22.3 ± 0.8°), confirming that chain A is significantly different. Comparisons of this structure with the symmetric open pore (PDBID 3zjz) tilt and swing values 140.1 and 26.7 , respectively, for all 4 chains, indicating the chain A of the partially open pore is more representative of the fully open conformation. This can be seen in Supplementary Figure S3 where in a superposition of the partially and fully open pores chain A of 4f4l shows the best overlay with it's equivalent in 3zjz.

Supplementary Table S3: Tilt and swing values for NavMs calculated usinf residue 30 to define the axis showing a comparison of partially open (chain A) (4f4l) and fully open (3zjz) (all chains) structures.

Structure	Helix Residues	Chain A		Chain B		Chain C		Chain D	
		θt	θs	θt	θs	θt	θs	θt	θs
4f4l	70-90	**141.0**	**27.2**	142.1	23.0	143.3	22.3	141.6	21.5
3zjz	70-90	**140.1**	**26.7**	140.2	26.7	140.1	26.7	140.2	26.7

Supplementary Figure S3: Partially (red) and full (green) open NavMs structures overlaid, with the chains letters as in Table S3. The helical axis for chain A is shown as the solid black line, and highlights that the A chains in both structures are the same, but the other chain are not. The superposition was performed using the Pymol "super" command. The pore axis is shown as a dotted line in the left panel and a circled cross in the right panel, which was produced by a 90 degree rotation of the figure in the left panel.

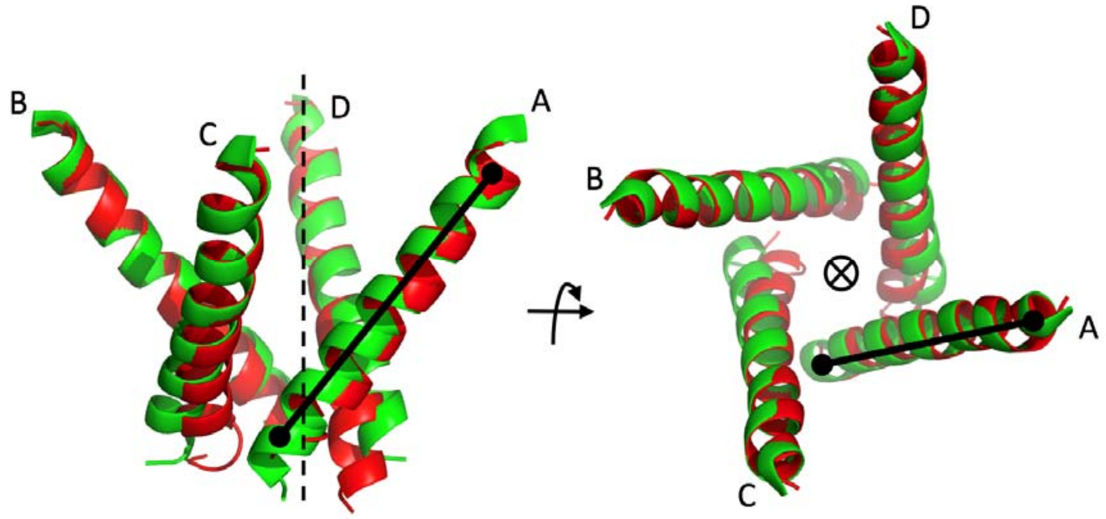

Supplementary Information References:

Alam,A. and Jiang,Y. (2009). Structural analysis of ion selectivity in the NaK channel. *Nat. Struct. Mol. Biol.*, 16, 35–41.

Bagneris,C., Decaen,P.G., Hall,B.A., Naylor,C.E., Clapham,D.E., Kay,C.W.M. and Wallace,B.A. (2013). Role of the C-terminal domain in the structure and function of tetrameric sodium channels. *Nat. Comms.*, 4, 2465.

McCusker,E.C., Bagneris,C., Naylor,C.E., Cole,A.R., D'Avanzo,N., Nichols,C.G. and Wallace,BA. (2012). Structure of a bacterial voltage-gated sodium channel pore reveals mechanisms of opening and closing. *Nat Comms.*, 3, 1102–1102.

Sauguet,L., Fourati,Z., Prange,T., Delarue,M. and Colloc'h,N. (2016). Structural basis for xenon inhibition in a cationic pentameric ligand-gated ion channel. *Plos One*, 11, e0149795.

Shi,N., Ye,S., Alam,A., Chen,L. and Jiang,Y. (2006). Atomic structure of a Na+- and K+-conducting channel. *Nature*, 440, 570–574.

Smart,O.S., Neduvelil,JG., Wang,X., Wallace,BA. and Sansom, M.S.P. (1996). HOLE: a program for the analysis of the pore dimensions of ion channel structural models. *J Mol. Graphics*, 14, 354-360.
